# Supplementary material for: Oncogenic drivers dictate immune control of acute myeloid leukemia
Source: Nat Commun. 2023 Apr 14;14:2155. doi: 10.1038/s41467-023-37592-9 (PMC10104832; doi:10.1038/s41467-023-37592-9)
Supplement: Supplementary file 1 — Supplementary Information [file 41467_2023_37592_MOESM1_ESM.pdf]

Supplementary Information

Oncogenic drivers dictate immunological control of disease progression in acute myeloid leukemia.

Rebecca J. Austin*, Jasmin Straube*, Rohit Halder, Yashaswini Janardhanan, Claudia Bruedigam, Matthew Witkowski, Leanne Cooper, Amy Porter, Matthias Braun, Fernando Souza-Fonseca-Guimaraes, Simone A Minnie, Emily Cooper, Sebastien Jacquelin, Axia Song, Tobias Bald, Kyohei Nakamura, Geoffrey R. Hill, Iannis Aifantis, Steven W. Lane[ϕ], Megan J. Bywater[ϕ].

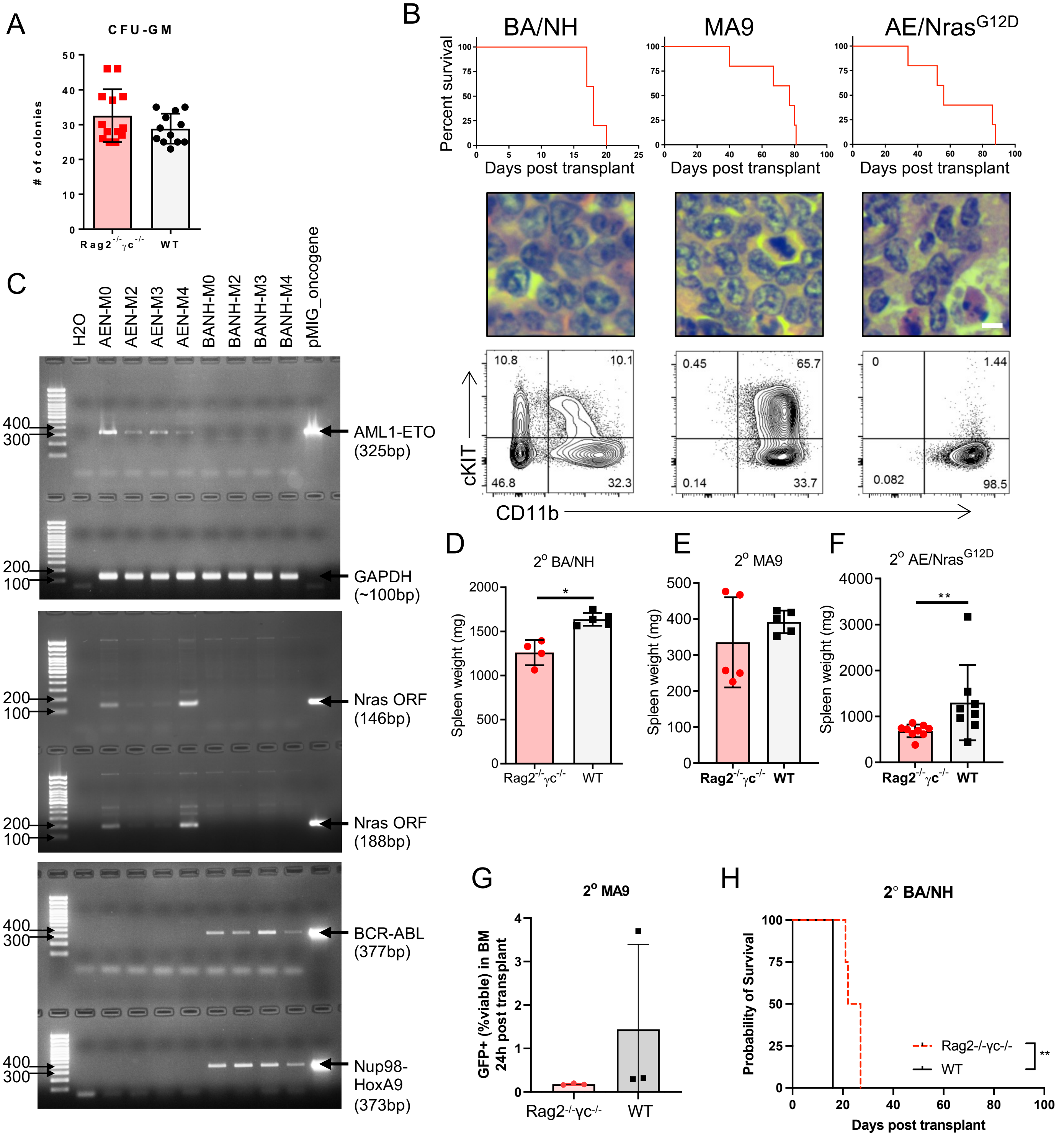

Supplementary Figure 1

Supplementary Figure 1: Oncogene specificity dictates AML immunogenicity. (A) Number of CFU-GM colonies grown in methylcellulose from wild type (WT, n=12) and Rag2$^{-/-}$γc$^{-/-}$ (n=13) mice. Data are presented as mean values +/- SD. (B) Kaplan-Meier curves for Rag2$^{-/-}$γc$^{-/-}$ primary recipients transplanted with Rag2$^{-/-}$γc$^{-/-}$ HSPCs subsequent to retroviral transduction with constructs expressing the oncogenes BCR-ABL and NUP98-HOXA9 (BA/NH, n=5), MLL-AF9 (MA9, n=5) or AML1-ETO and Nras^{G12D} (AE/Nras^{G12D}, n=5) (top). Cell morphology of secondary BA/NH, MA9 and AE/Nras^{G12D} AML in H&E stained spleens of Rag2$^{-/-}$γc$^{-/-}$ recipients (middle, scale bar = 20μm). Cell surface expression by flow cytometry of CD11b and cKIT on GFP+ primary BA/NH, MA9 and AE/Nras^{G12D} AMLs passaged through Rag2$^{-/-}$γc$^{-/-}$ recipients (bottom). Transduction performed once, cell morphology and surface marker expression on a representative example of the whole cohort. (C) PCR using gDNA isolated from GFP+ AML cells from BA/NH and AE/ Nras^{G12D} primary recipients and primers spanning the AML1-ETO, BCR-ABL and NUP98-HOXA9 breakpoints and exon 4 and 5 in Nras. Amplification of GAPDH was used as a positive control. PCR performed once, each lane represents a biologically independent animal. Spleen weights of secondary recipients transplanted with primary (D) BA/NH (Rag2$^{-/-}$γc$^{-/-}$ n=4, WT n=5, p=0.0159), (E) MA9 (Rag2$^{-/-}$γc$^{-/-}$ n=5, WT n=5), and (F) AE/Nras^{G12D} (Rag2$^{-/-}$γc$^{-/-}$ n=9, WT n=8, p=0.0079). Data are presented as mean values +/- SD. (G) Percentage of GFP+ 2° MA9 AML cells in BM 24h post transplant (n=3 per group). Data are presented as mean values +/- SD. (H) Kaplan-Meier curves comparing survival between Rag2$^{-/-}$γc$^{-/-}$ (n=4) and WT (n=5) secondary recipients transplanted with 1,000 1° BA/NH AML cells (p=0.0047). Each point represents a biologically independent animal. Two-tailed Mann-Whitney test for comparison

between two groups (D-F). Mantel-Cox test for comparison of Kaplan-Meier curves, *$p < .05$, ** $p < 0.01$. Source data are provided as a Source Data file.

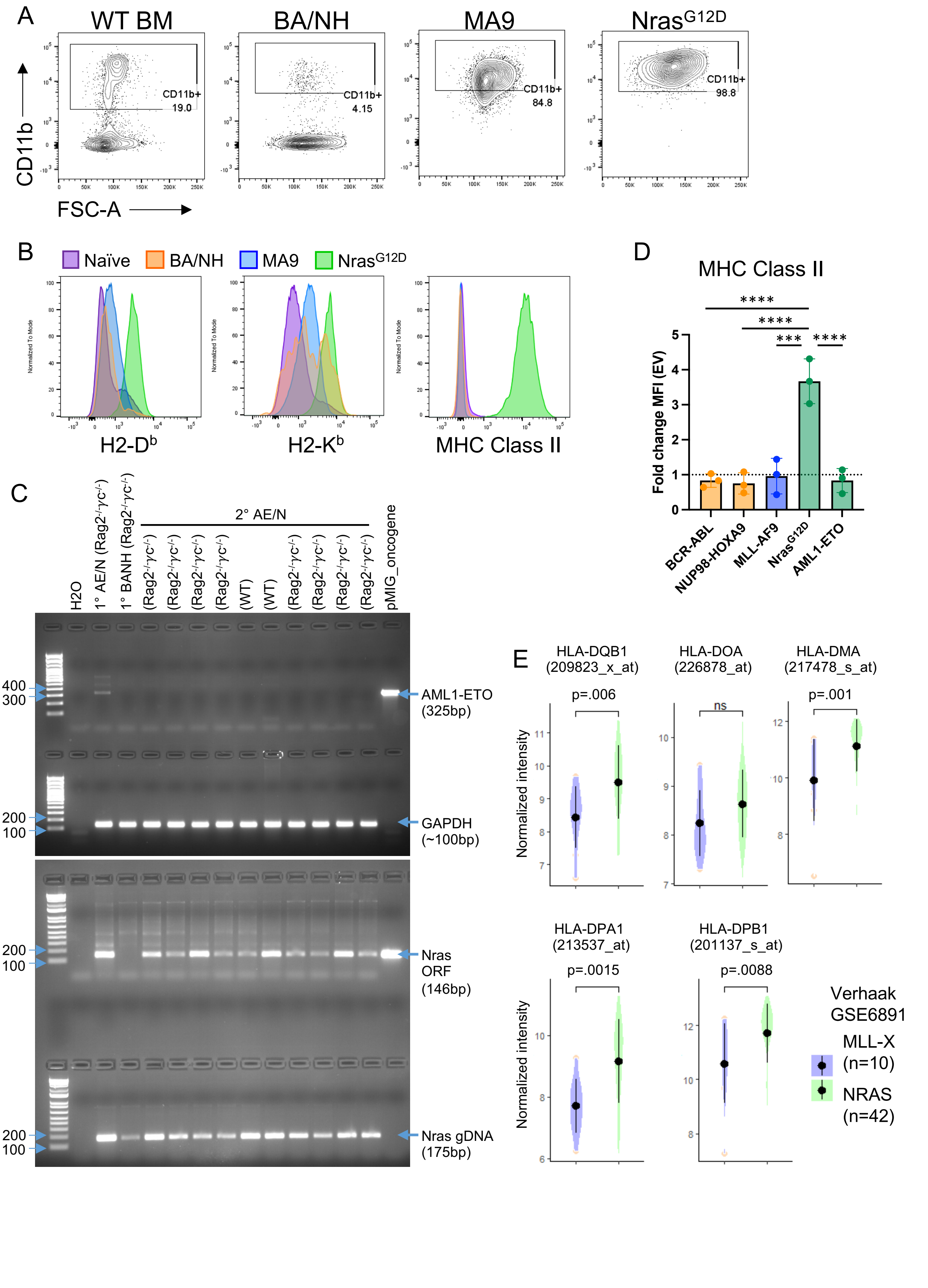
A
WT BM
BA/NH
MA9
Nras^{G12D}
CD11b+ 19.0
CD11b+ 4.15
CD11b+ 84.8
CD11b+ 98.8
CD11b
FSC-A
B
Naïve
BA/NH
MA9
Nras^{G12D}
H2-D^b
H2-K^b
MHC Class II
Normalized To Mode
C
H2O
1° AE/N (Rag2$^{-/-}\gamma c^{-/-}$)
1° BANH (Rag2$^{-/-}\gamma c^{-/-}$)
2° AE/N
(Rag2$^{-/-}\gamma c^{-/-}$)
(WT)
pMIG_oncogene
400
300
AML1-ETO (325bp)
200
100
GAPDH (~100bp)
Nras ORF (146bp)
Nras gDNA (175bp)
D
MHC Class II
Fold change MFI (EV)
****
***
BCR-ABL
NUP98-HOXA9
MLL-AF9
Nras^{G12D}
AML1-ETO
E
HLA-DQB1 (209823_x_at)
HLA-DOA (226878_at)
HLA-DMA (217478_s_at)
p=.006
ns
p=.001
Normalized intensity
HLA-DPA1 (213537_at)
HLA-DPB1 (201137_s_at)
p=.0015
p=.0088
Verhaak GSE6891
MLL-X (n=10)
NRAS (n=42)

Supplementary Figure 2: Oncogene specificity influences the immunogenicity of AML cells. (A) Gating strategy for monocytes/granulocytes (CD11b+). (B) Histograms comparing expression of $H2D^b$, $H2K^b$ and MHC Class II (IA/E) on CD11b+ cells from naïve mice and on the cell surface of GFP+ BA/NH, MA9 and $Nras^{G12D}$ cells. (C) PCR using gDNA isolated from GFP+ AML cells from BA/NH and AE/$Nras^{G12D}$ primary and secondary recipients and primers spanning the AML1-ETO breakpoint and exon 4 and 5 in Nras. Amplification of GAPDH was used as a positive control. PCR performed once, each lane represents a biologically independent animal. (D) Median fluorescence intensity (MFI) of MHC Class II (IA/E) on cell surface of CD11b+ GFP+ cultured hematopoietic stem and progenitor cells (HSPCs) isolated from $Rag2^{-/-}\gamma c^{-/-}$ bone marrow, 72hrs post transduction with individual retroviral constructs expressing the oncogenes listed on the x-axis or an empty vector (EV) control (p=0.0001(MLL-AF9 vs $Nras^{G12D}$)). MFI expressed as fold change EV control, n=3 independent HPSC donors and transductions, data are presented as mean values +/- SD. (E) Microarray derived gene expression of HLA-DQB1, HLA-DOA, HLA-DMA, HLA-DPA1 and HLA-DPB1 from bulk PB/BM of n=10 MLL-X and 42 mutant NRAS AML patients[1]. Data are presented as mean values +/- SD. One-way ANOVA with Tukey's multiple testing correction (D), Mann-Whitney test for pairwise comparisons between groups (E). * p <0.05, ** p < 0.01, *** p <0.001, **** p <0.0001. Source data are provided as a Source Data file.

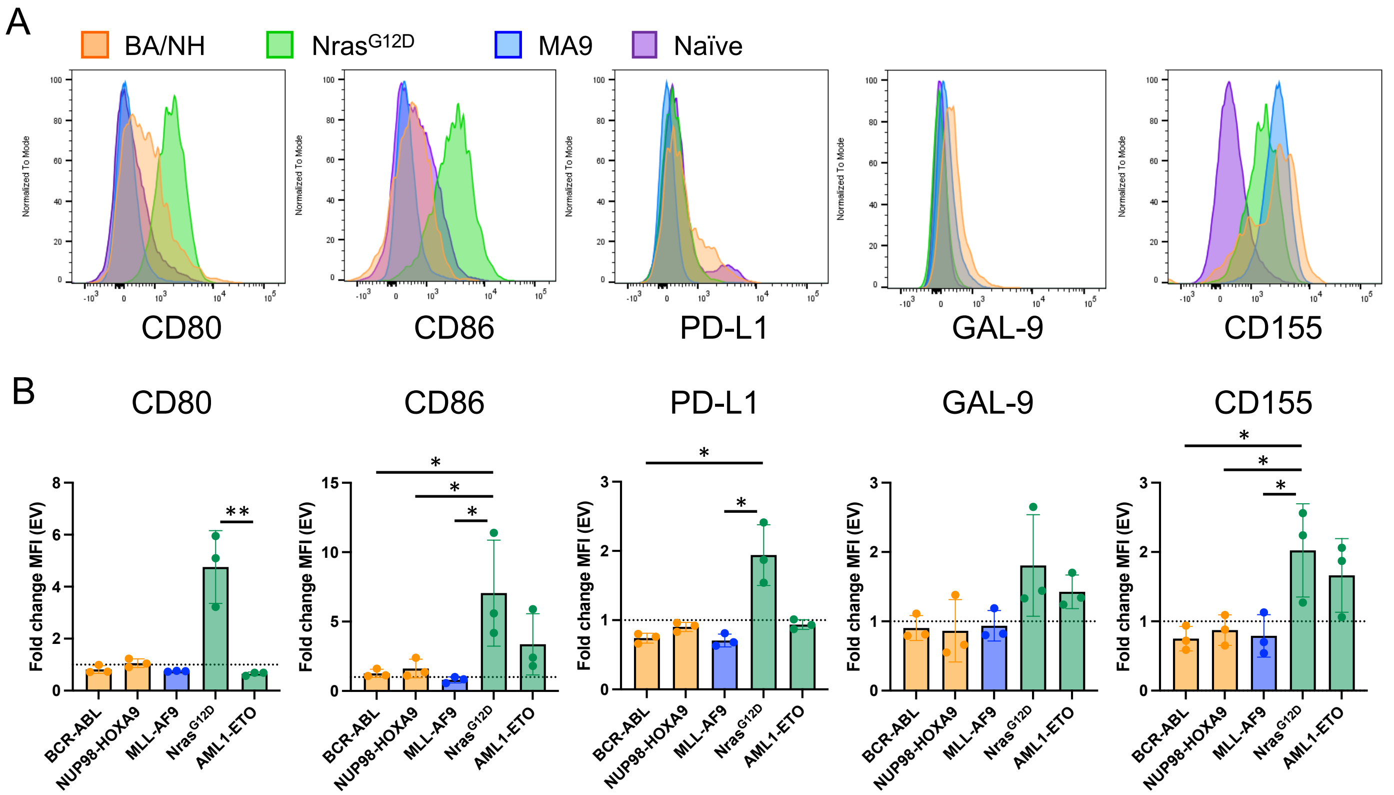

Supplementary Figure 3

Supplementary Figure 3: Oncogene specificity influences the immunogenicity of AML cells. (A) Histograms comparing expression of CD80, CD86, PD-L1, GAL-9 and CD155 on CD11b+ cells from naïve mice and on the cell surface of GFP+ BA/NH, MA9 and $Nras^{G12D}$ cells. (B) Median fluorescence intensity (MFI) of CD80, CD86, PD-L1, GAL-9 and CD155 on cell surface of CD11b+ GFP+ cultured hematopoietic stem and progenitor cells (HSPCs) isolated from $Rag2^{-/-}\gamma c^{-/-}$ bone marrow, 72hrs post transduction with individual retroviral constructs expressing the oncogenes listed on the x-axis or an empty vector (EV) control. MFI expressed as fold change EV control, n=3 independent HPSC donors and transductions, data are presented as mean values +/- SD. One-way ANOVA with Tukey's multiple testing correction (B: CD86, GAL-9, CD155), Kruskal-Wallis test (B: CD80, PD-L1). * $p < 0.05$, ** $p < 0.01$, *** $p < 0.001$, **** $p < 0.0001$. Source data are provided as a Source Data file.

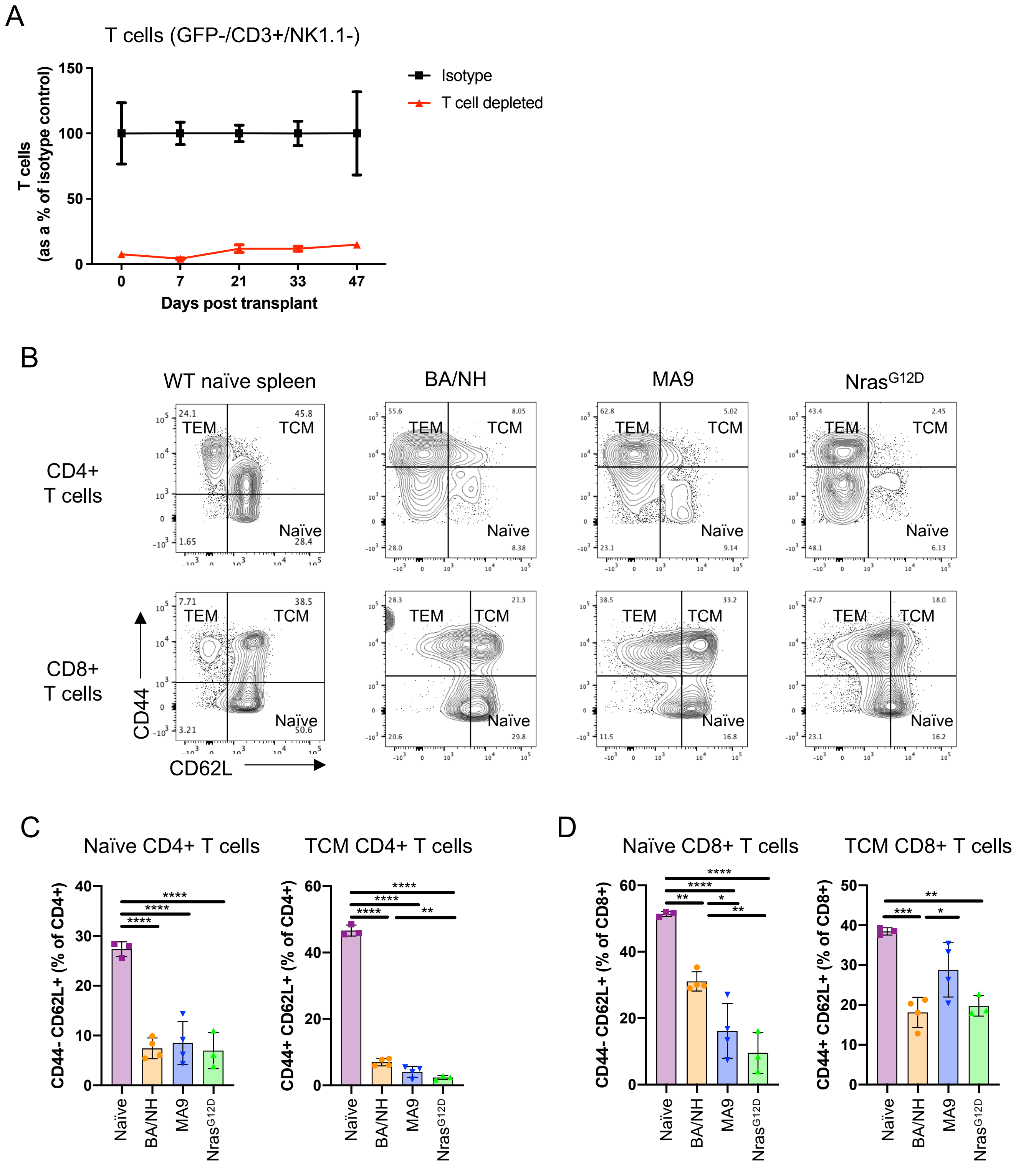

Supplementary Figure 4

Supplementary Figure 4: Oncogene specificity influences the type of immune response to AML cells. (A) Frequency of T cells (GFP-/CD3+/NK1.1) as a percentage of GFP- mononuclear cells in the peripheral blood of wild type mice treated with T cell depleting antibodies (anti-CD4/anti-CD8, n=5) or isotype control (n=5) and transplanted with MA9 AML, as expressed as a percentage of the average for the isotype control per timepoint. Data are presented as mean values +/- SD. (B) Gating strategy and representative flow plots for naïve (CD44- CD62L+), T effector memory (TEM, CD44+ CD62L-) and central memory (TCM, CD44+ CD62L+) CD4+ and CD8+ T cells from naïve mice and BA/NH, MA9 and $Nras^{G12D}$ AML recipients. (C) Frequency of naïve and TCM (p=0.0053(BA/NH vs $Nras^{G12D}$) CD4+ T cells and (D) naïve (p=0.0033(Naïve vs BA/NH), p=0.0153(BA/NH vs MA9), p=0.0022(BA/NH vs $Nras^{G12D}$)) and TCM (p=0.0006(Naïve vs BA/NH), p=0.0021(Naïve vs $Nras^{G12D}$), p=0.029(BA/NH vs MA9)) CD8+ T cells in the spleens of naïve wild type C57BL/6J mice (n=3) and BA/NH (n=4), MA9 (n=4) and $Nras^{G12D}$ (n=3) AML recipients. Data are presented as mean values +/- SD. One-way ANOVA with Tukey's p-value adjustment. * p <0.05, ** p < 0.01, *** p <0.001, **** p <0.0001. Each point represents a biologically independent animal. Source data are provided as a Source Data file.

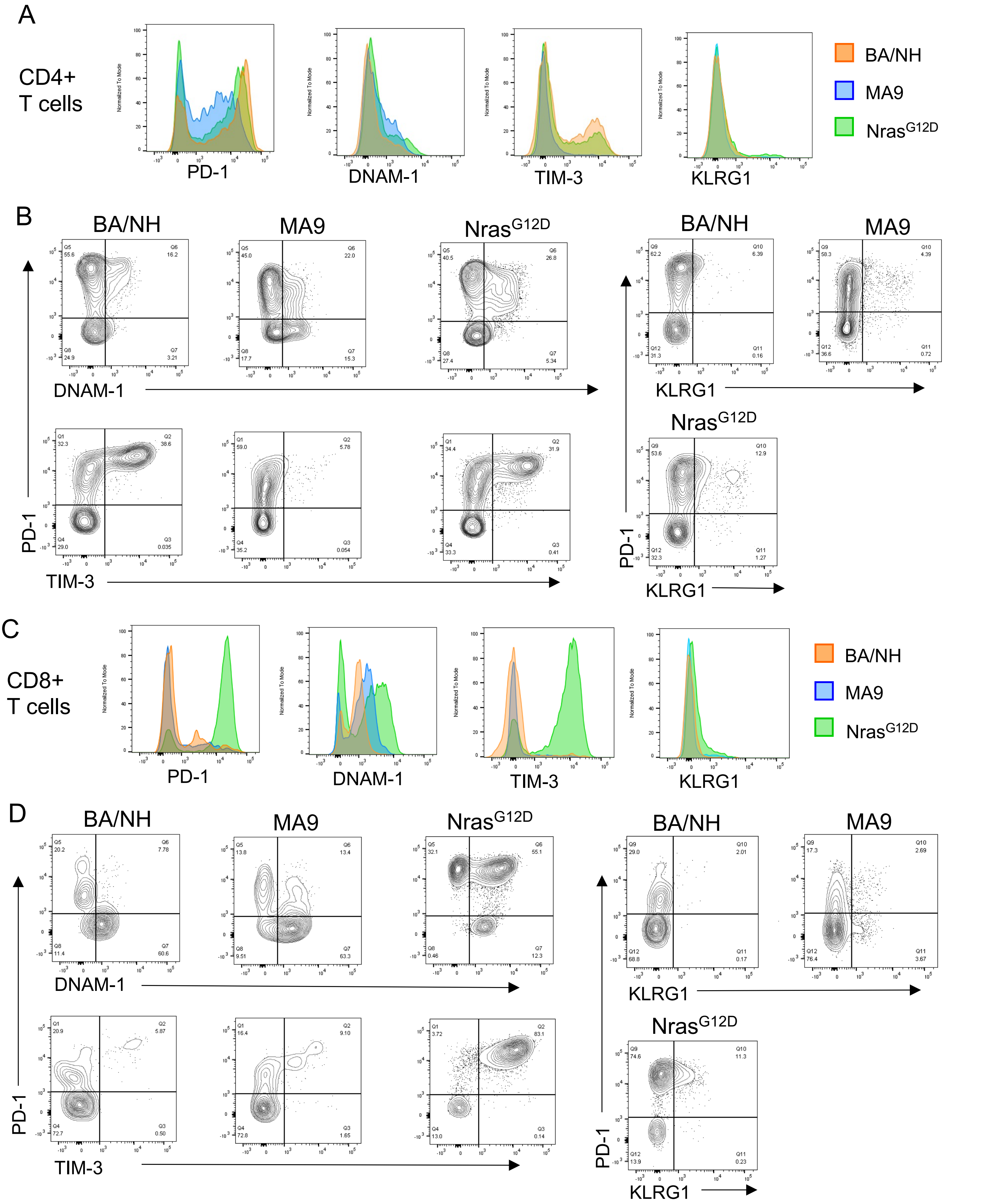

Supplementary Figure 5

Supplementary Figure 5: Oncogene specificity influences the type of immune response to AML cells. (A) Representative histograms displaying expression of PD-1, DNAM-1, TIM-3, and KLRG1 on CD4+ T cells from BA/NH, MA9 and $Nras^{G12D}$ immunocompetent recipients. (B) Representative flow plots of CD4+ T cells from BA/NH, MA9 and $Nras^{G12D}$ immunocompetent recipients showing co-expression of PD-1 and DNAM-1, PD-1 and TIM-3, and PD-1 and KLRG1. (C) Representative histograms displaying expression of PD-1, DNAM-1, TIM-3 and KLRG1 on CD8+ T cells from BA/NH, MA9 and $Nras^{G12D}$ immunocompetent recipients. (D) Representative flow plots of CD8+ T cells from BA/NH, MA9 and $Nras^{G12D}$ immunocompetent recipients showing co-expression of PD-1 and DNAM-1, PD-1 and TIM-3, and PD-1 and KLRG1.

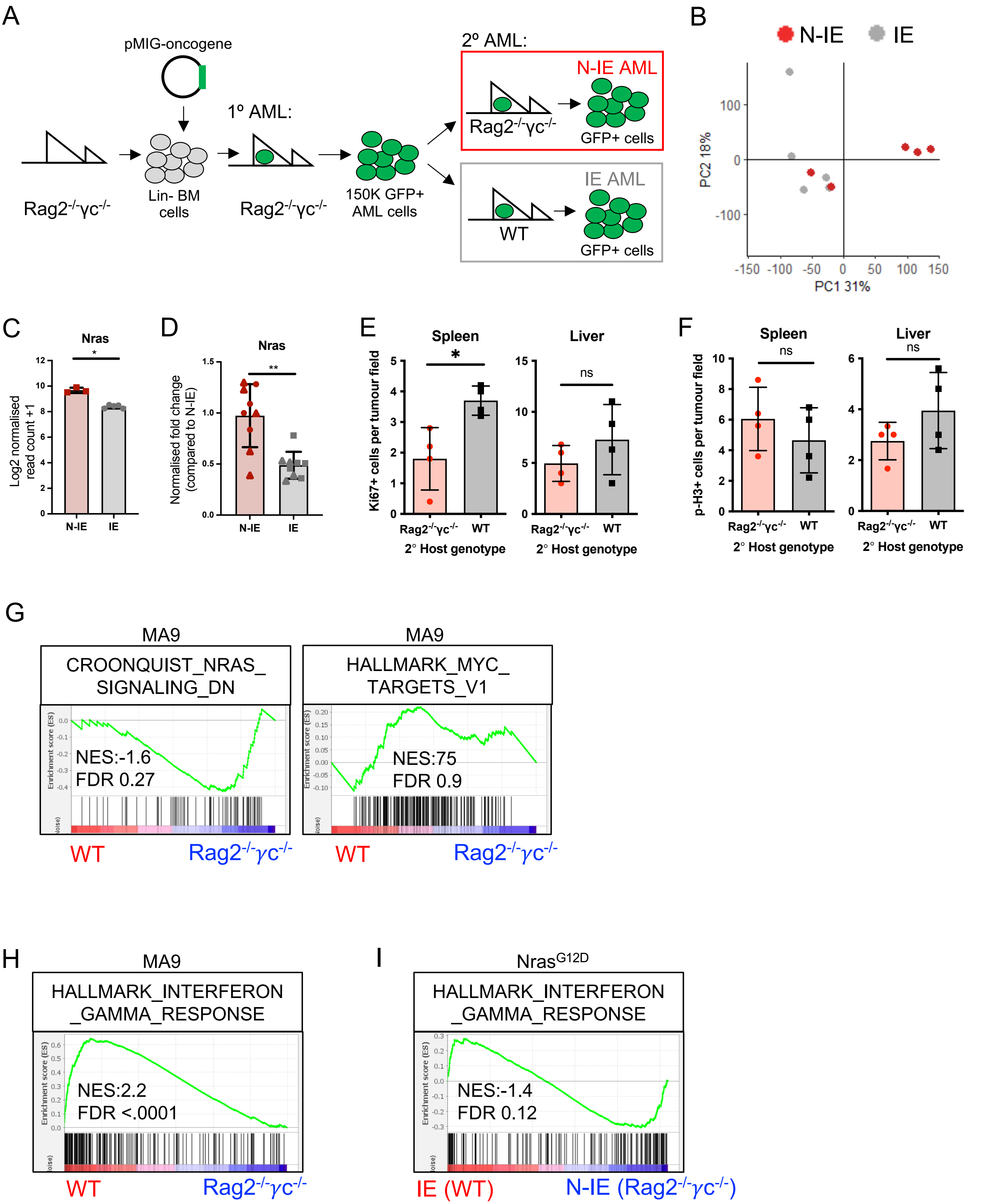

Supplementary Figure 6

Supplementary Figure 6: Nras^{G12D} AML cells escape immunologic control through immunoediting. (A) Experimental schema for the generation of immunoedited (IE) and non-immunoedited (N-IE) Nras^{G12D} cells for RNA sequencing. (B) Principal component analysis of RNA sequencing data generated from GFP+ Nras^{G12D} AML cells isolated from either immunocompetent WT (IE, n=5) or immunodeficient Rag2$^{-/-}$γc$^{-/-}$ (N-IE, n=5) recipients. (C) Quantification of Nras normalized read-count in N-IE (n=3) and IE (n=5) Nras^{G12D} cells (p=0.0357). (D) Quantification of Nras gene expression by qPCR in N-IE (n=9) and IE (n=9) Nras^{G12D} cells (p=0.0017). Data are presented as mean values +/- SD. (E) Quantification of Ki67 immunohistochemistry on tumour-bearing regions of the spleen and liver isolated from either Rag2$^{-/-}$γc$^{-/-}$ or WT secondary recipients of Nras^{G12D} AML. (n=4 independent recipients, each data point represents the mean of 5 separate fields, p=0.0286(Spleen)). Data are presented as mean values +/- SD. (F) Quantification of phosphorylated histone H3 (p-H3) immunohistochemistry on tumour-bearing regions of the spleen and liver isolated from either Rag2$^{-/-}$γc$^{-/-}$ or WT secondary recipients of Nras^{G12D} AML. (n=4 independent recipients, each data point represents the mean of 5 separate fields). Data are presented as mean values +/- SD. (G) Lack of enrichment of genes correlating with down-regulation of Nras signaling and upregulation of Myc targets in MA9 AML passaged in WT (n=4) compared to Rag2$^{-/-}$γc$^{-/-}$ (n=5) recipients, as determined from RNA-sequencing of GFP+ AML cells. (H) Enrichment of genes correlating with an interferon gamma response in MA9 AML passaged in WT (n=4) compared to Rag2$^{-/-}$γc$^{-/-}$ (n=5) recipients, as determined from RNA-sequencing of GFP+ AML cells. (I) Enrichment of genes correlating with an interferon gamma response in non-immunoedited (N-IE) Nras^{G12D} AML, as

determined from RNA-sequencing of GFP+ AML cells isolated from either immunocompetent WT (IE, n=5) or immunodeficient Rag2$^{-/-}$γc$^{-/-}$ (N-IE, n=5) recipients. Two-tailed Mann-Whitney test for comparison between two groups (C, D, E, F). Each point represents a biologically independent animal. * $p < 0.05$, ** $p < 0.01$. Source data are provided as a Source Data file.

Supplementary Table 1: Sequence over breakpoint for AML fusion oncogenes.

Fusion Oncogene	Breakpoint	Sequencing Primer	Sequencing product
AML1-ETO (RUNX1-RUNX1T1)	Exon6 – Exon3	AML-ES 5'GAGGGAAAAG CTTCACTCTG3'	CCTACCACAGAGCCATCAAAATCACAGTGGATGGGCCCCGAGAACCTCGAAATCGTACTG AGAAGCACTCCACAATGCCAGACTCACCTGTGGATGTGAAGACGCAATCTAGGCTGACTC CTCCAACAATGCCACCTCCCCCAACTACTCAAGGAGCTCCAAGAACCAGTTCATTTACAC CGACAACGTTAACTAATGGCACGAGCCATTCTCCTACAGCCTTGAATGGCGCCCCCTCAC CACCCAATGGCTTCAGCAATGGGCCTTCCTCTTCTTCCTCCTCCTCTCTGGCTAATCAACA GCTGCCCCCAGCCTGTGGTGCCAGGCAACTCAGCAAGCTGAAAAGGTTCCTTACTACCCT GCAGCAGTTTGGCAATGACATTTCACCCGAGATAGGAGAAAGAGTTCGCACCCTCGTTCT GGGACTAGTGAACTCCACTTTGACAATTGAAGAATTTCATTCCAAACTGCAAGAAGCTACT AACTTCCCACTGAGACCTTTTGTCATCCCATTTTTGAAGGCCAACTTGCCCCTGCTGCAGC GTGAGCTCCTCCACTGCGCAAGACTG
MLL-AF9 (KMT2A-MLLT3)	Exon10 – Exon9	MLL-F1<br><br>5'CGCCTCAGCC ACCTACTACAG3'	TCCTAGTGAGCCCAAGAAAAAGCAGCCTCCACCACCAGAATCAGGTCCAGAGCAGAGCA AACAGAAAAAAGTGGCTCCCCGCCCAAGTATCCCTGTAAAACAAAAACCAAAAGAAAAGG AAAAACCACCTCCGGTCAATAAGCAGGAGAATGCAGGCACTTTGAACATCCTCAGCACTC TCTCCAATGGCAATAGTTCTAAGCAAAAAATTCCAGCAGATGGAGTCCACAGGATCAGAG TGGACTTTAAGGAAGACTGTGAAGCAGAAAATGTGTGGGAGATGGGAGGCTTAGGGATC CTTGAAGTGAAAAGTCCAATAAAGCAAAGCAAATCAGATAAGCAAATAAAGAATGGTGAAT GTGACAAGGCATACCTAGATGAACTGGTAGAGCTTCACAGAAGGTTAATGACATTGAGAG AAAGACACATTCTGCAGCAGATCGTGAACCTTATAGAAGAAACTGGACACTTTCATATCAC AAACACAACATTTGATTTTGATCTTTGCTCGCTGGACAAAACCACAGTCCGTAAACTACAG AGTTACCTGGAAACATCTGGAACATCCTGAG
BCR-ABL1	Exon14-Exon2	BCRP210-B2-C 5' CAGATGCTGAC CAACTCGTGT3'	GATGATGAGTCTCCGGGGCTCTATGGGTTTCTGAATGTCATCGTCCACTCAGCCACTGGA TTTAAGCAGAGTTCAAAAGCCCTTCAGCGGCCAGTAGCATCTGACTTTGAGCCTCAGGGT CTGAGTGAAGCCGCTCGTTGGAACTCCAAGGAAAACCTTCTCGCTGGACCCAGTGAAAAT GACCCCAACCTTTTCGTTGCACTGTATGATTTTGTGGCCAGTGGAGATAACACTCTAAGCA TAACTAAAGGTGAAAAGCTCCGGGTCTTAGGCTATAATCACAATG

NUP98-HOXA9	Exon12 – Exon1	NUP98 forward-3 5’GCACAAATACCAGTGGGAATA 3’	GGACTGGGCTTGGTGCAGGATTTGGAACAGCTCTTGGTGCTGGACAGGCATCTTTGTTTGGGAACAACCAACCTAAGATTGGAGGGCCTCTTGGTACAGGAGCCTTTGGGGCCCCTGGATTTAATACTACGACAGCCACTTTGGGCTTTGGAGCCCCCCAGGCCCCAGTAGTTGATAGAGAAAAACAACCCAGCGAAGGCGCCTTCTCTGAAAACAATGCTGAGAATGAGAGCGGCGGAGACAAGCCCCCCATCGATCCCAATAACCCAGCAGCCAACTGGCTTCATGCGCGCTCCACTCGGAAAAAGCGGTGCCCCTATACAAAACACCAGACCCTGGAACTGGAGAAAGAGTTTCTGTTCAACATGTACCTCACCAGGGACCGCAGGTACGAGGTGGCTCGACTGCTCAACCTCACCGAGAGGCAGGTCAAGATCTGGTTCCAGAACCGCAGGATGAAAATGAAGAAAATCAACAAAGACCGAGCAAAAGACGAGTGATGCCATTTGGGCTTATTTAGA

Supplementary Table 2: Flow cytometry antibodies

Marker	Clone	Manufacturer	Dilution
TCR-β	H57-597	Biolegend (109227)	1:200
CD4	RM4-5	Biolegend (100525)	1:200
CD8	53-6.7	Biolegend (100747)	1:200
H-2D^b	28-14-8	BD Biosciences (553601)	1:200
H2-K^b	AF6-88-5	BD Biosciences (550550)	1:200
CD80	16-10A1	eBioscience (17-0801-82)	1:100
CD86	GL-1	Biolegend (105037)	1:100
CD155	TX56	Biolegend (131510)	1:100
PD-L1	10F.9G2	Biolegend (124313)	1:100
TIM-3	RMT3-23	eBioscience (119721)	1:100
MHC Class II	M5/114.15.2	eBioscience (17-5321-82)	1:200
GAL-9	108A.2	Biolegend (137903)	1:200
CD44	IM7	Biolegend (103023)	1:200
CD62L	MEL-14	Biolegend (104426)	1:200
PD-1	29F.1A12	Biolegend (135219)	1:200
DNAM-1	10E5	Biolegend (128811)	1:200

KLRG1	2F1	Biolegend (138415)	1:200

References

1. Verhaak, R.G., et al., *Prediction of molecular subtypes in acute myeloid leukemia based on gene expression profiling.* Haematologica, 2009. **94**(1): p. 131-4.
