# Supplementary material for: Oncogenic drivers dictate immune control of acute myeloid leukemia
Source: Nat Commun. 2023 Apr 14;14:2155. doi: 10.1038/s41467-023-37592-9 (PMC10104832; doi:10.1038/s41467-023-37592-9)
Supplement: Supplementary file 3 — Reporting Summary [file 41467_2023_37592_MOESM3_ESM.pdf]

nature portfolio

Corresponding author(s): Megan Bywater, Steven W. Lane

Last updated by author(s): Mar 2, 2023

Reporting Summary

Nature Portfolio wishes to improve the reproducibility of the work that we publish. This form provides structure for consistency and transparency in reporting. For further information on Nature Portfolio policies, see our Editorial Policies and the Editorial Policy Checklist.

Statistics

For all statistical analyses, confirm that the following items are present in the figure legend, table legend, main text, or Methods section.

n/a	Confirmed	
☐	☒	The exact sample size (*n*) for each experimental group/condition, given as a discrete number and unit of measurement
☐	☒	A statement on whether measurements were taken from distinct samples or whether the same sample was measured repeatedly
☐	☒	The statistical test(s) used AND whether they are one- or two-sided<br>*Only common tests should be described solely by name; describe more complex techniques in the Methods section.*
☐	☒	A description of all covariates tested
☐	☒	A description of any assumptions or corrections, such as tests of normality and adjustment for multiple comparisons
☐	☒	A full description of the statistical parameters including central tendency (e.g. means) or other basic estimates (e.g. regression coefficient) AND variation (e.g. standard deviation) or associated estimates of uncertainty (e.g. confidence intervals)
☐	☒	For null hypothesis testing, the test statistic (e.g. *F*, *t*, *r*) with confidence intervals, effect sizes, degrees of freedom and *P* value noted<br>*Give P values as exact values whenever suitable.*
☒	☐	For Bayesian analysis, information on the choice of priors and Markov chain Monte Carlo settings
☒	☐	For hierarchical and complex designs, identification of the appropriate level for tests and full reporting of outcomes
☐	☒	Estimates of effect sizes (e.g. Cohen's *d*, Pearson's *r*), indicating how they were calculated

Our web collection on statistics for biologists contains articles on many of the points above.

Software and code

Policy information about availability of computer code

Data collection	FACS Diva Software (version 8.0.1), Hemavet DMS Capture (version 1.0.0), NextSeq System Suite (version 2.1.2 - RNAseq), QuantStudio Real-Time PCR System (version 1.3), Scanscope (version 102.0.7.5)
Data analysis	Cutadapt (version 1.11), RSEM (version 1.2.30), STAR (version 2.5.2a), edgeR (version 3.28.1), GSEA (version 4.1.0), GSVA (version 1.34.0), GraphPad Prism (version 7.02), FlowJo (version 10.8.0), Image scope (version 12.4), Primer3web (version 4.1.0)

For manuscripts utilizing custom algorithms or software that are central to the research but not yet described in published literature, software must be made available to editors and reviewers. We strongly encourage code deposition in a community repository (e.g. GitHub). See the Nature Portfolio guidelines for submitting code & software for further information.

Data

Policy information about availability of data

All manuscripts must include a data availability statement. This statement should provide the following information, where applicable:

- Accession codes, unique identifiers, or web links for publicly available datasets
- A description of any restrictions on data availability
- For clinical datasets or third party data, please ensure that the statement adheres to our policy

Generated bulk RNA sequencing data are available from GEO with accession numbers GSE164951 and GSE207316. Public available AML Microarray array data have accession GSE6891. Public available AML and healthy single cell CITE and RNASeq data have accession GSE185381. RNAseq reads from murine experiments were mapped to mouse genome build GRCm38 with ensembl v70 gene model downloaded from ensembl on 11 June 2015.

Human research participants

Policy information about studies involving human research participants and Sex and Gender in Research.

Reporting on sex and gender	n/a
Population characteristics	n/a
Recruitment	n/a
Ethics oversight	n/a

Note that full information on the approval of the study protocol must also be provided in the manuscript.

Field-specific reporting

Please select the one below that is the best fit for your research. If you are not sure, read the appropriate sections before making your selection.

[x] Life sciences   [ ] Behavioural & social sciences   [ ] Ecological, evolutionary & environmental sciences

For a reference copy of the document with all sections, see nature.com/documents/nr-reporting-summary-flat.pdf

Life sciences study design

All studies must disclose on these points even when the disclosure is negative.

Sample size	No statistical method was used to predetermine sample sizes used for animal experiments. The sample size was determined based on prior experience with the experimental models used. At least three biologically independent replicates per group were collected to generate statistical testing.
Data exclusions	Data was excluded on very rare occasions when abnormal output could be clearly attributed to poor sample quality or pipetting error.
Replication	Figure 1, Supp Fig 1D-F: BA/NH demonstrating data from one experiment with findings replicated in an independent experiment where fewer cells were transplanted. MA9 and AE/NrasG12D demonstrating pooled data from two independent experiments.<br>Figure 2A and E, 3E-F and Supp Fig 2A, 3A, 4B-D, 5A-D: analysis performed once on samples generated across at least 2 independent experiments.<br>Figure 2F-G: demonstrating data from the transplant of one AML into 6 biologically independent recipients per genotype<br>Figure 3A: demonstrating data from the transplant of one AML into 4-5 biologically independent recipients per treatment. A repeat experiment was also performed that replicated the result.<br>Figure 3B: demonstrating pooled data from two independent experiments.<br>Figure 3C-D: Representative data from two experiments showing the same result.<br>Figure 4B-C: Demonstrating data from one experiment with findings replicated in an independent experiment.<br>Figure 4D-E: Demonstrating pooled data from analysis performed on 2 separate occasions.<br>Figure 4G: Demonstrating pooled data from two independent experiments.<br>Figure 5A-C and Supp Fig6B, 6C, 6I: demonstrating the data from one AML into 5 biologically independent recipients per genotype.<br>Figure 5E: demonstrating the data from one AML into 5 biologically independent recipients per genotype.<br>Figure 5F: demonstrating the data from one AML into 5 biologically independent recipients per genotype. A repeat experiment was also performed that replicated the result.<br>Supplementary Fig 1A: Experiment was performed once, demonstrating data points derived from 12 biologically independent mice.<br>Supplementary Fig 1B: Transduction was performed once, demonstrating data points from 5 biologically independent recipients per genotype.<br>Supplementary Fig 1C: PCR performed once, demonstrating data from biologically independent recipients.<br>Supplementary Fig 1G: Experiment was performed once, demonstrating data points derived from 3 biologically independent mice. Result was supported by data presented in Figure 1 (BA/NH).

Supplementary Fig 2C: PCR performed once, demonstrating data from biologically independent recipients.
Supplementary Fig 2D and 3B: Demonstrating pooled data from analysis performed on 3 separate occasions using 3 independent donors and transductions.
Supplementary Fig 4A: Representative data from two experiments showing the same result.
Supplementary Fig 6D: Demonstrating pooled data from two independent experiments.
Supplementary Fig 6E-F: Analysis was performed once, demonstrating data points derived from 4 biologically independent mice. Result supported by data presented in Figure 4B.

Randomization	For studies involving the transplantation of different AMLs, recipient mice were assigned to groups at random whilst ensuring that all groups were matched in terms of age and gender. For studies involving the treatment of mice transplanted with the same AML with either vehicle or anti-PD-1, mice were randomly assigned to treatment cages prior to transplant, ensuring that groups were matched for both gender and age.
Blinding	The investigators were not blinded to allocation during experiments and outcome assessment. All samples were processed either simultaneously or in parallel in all experiments.

Reporting for specific materials, systems and methods

We require information from authors about some types of materials, experimental systems and methods used in many studies. Here, indicate whether each material, system or method listed is relevant to your study. If you are not sure if a list item applies to your research, read the appropriate section before selecting a response.

Materials & experimental systems

n/a	Involved in the study
☐	☒ Antibodies
☐	☒ Eukaryotic cell lines
☒	☐ Palaeontology and archaeology
☐	☒ Animals and other organisms
☒	☐ Clinical data
☒	☐ Dual use research of concern

Methods

n/a	Involved in the study
☒	☐ ChIP-seq
☐	☒ Flow cytometry
☒	☐ MRI-based neuroimaging

Antibodies

Antibodies used	TCR-β (H57-597) Biolegend (109227), CD4 (RM4-5) Biolegend (100525), CD8 (53-6.7) Biolegend (100747), H-2Db (28-14-8) BD Biosciences (553601), H2-Kb (AF6-88-5) BD Biosciences (550550), CD80 (16-10A1) eBioscience (17-0801-82), CD86 (GL-1) Biolegend (105037), CD155 (4.24.3) Biolegend (cat#), PD-L1 (10F.9G2) Biolegend (124313), TIM-3 (RMT3-23) eBioscience (119721), MHC Class II (M5/114.15.2) eBioscience (17-5321-82), GAL-9 (108A.2) Biolegend (137903), CD44 (IM7) Biolegend (103023), CD62L (MEL-14) Biolegend (104426), PD-1 (29F.1A12) Biolegend (135219), KLRG1 (2F1) Biolegend (138415), DNAM-1 (10E5) Biolegend (128811), control IgG (HRPN) Bio-X-Cell (BE0088), anti-CD4 (GK1.5) Bio-X-Cell (BE0003-1), anti-CD8b (53.5.8) Bio-X-Cell (BE0223), anti-PD-1 (RMP1-14) Bio-X-Cell (BE0146).
Validation	TCR-β (H57-597) Biolegend (109227): raised against affinity purified TCR from mouse DO-11.10 cells, validated by manufacturer by flow cytometric analysis of C57BL/6 splenocytes comparatively stained with anti-CD3 or an isotype control.<br>CD4 (RM4-5) Biolegend (100525): raised against BALB/c mouse thymocytes, validated by manufacturer by flow cytometric analysis of C57BL/6 splenocytes comparatively stained with an isotype control.<br>CD8 (53-6.7) Biolegend (100747): raised against mouse thymus or spleen, validated by manufacturer by flow cytometric analysis of C57BL/6 splenocytes stained in combination with anti-CD3.<br>H-2Db (28-14-8) BD Biosciences (553601): raised against C3H.SW mouse splenocytes, validated by manufacturer: Flow cytometry (Routinely Tested)<br>H2-Kb (AF6-88-5) BD Biosciences (550550): raised against Mouse C57BL/6 Splenocytes, validated by manufacturer: Flow cytometry (Routinely Tested)<br>CD80 (16-10A1) eBioscience (17-0801-82): validated by manufacturer: The 16-10A1 antibody has been tested by flow cytometric analysis of stimulated mouse splenocytes.<br>CD86 (GL-1) Biolegend (105037): raised against LPS-activated CBA/Ca mouse splenic B cells, validated by manufacturer by flow cytometric analysis of LPS-stimulated (3 days) C57BL/6 mouse splenocytes comparatively stained with an isotype control.<br>CD155 (TX56) Biolegend (131510): raised against CD155 transfectants, validated by manufacturer by flow cytometric analysis of C57BL/6 splenocytes and thymocytes<br>PD-L1 (10F.9G2) Biolegend (124313): validated by manufacturer by flow cytometric analysis of C57/B6 splenocytes comparatively stained with an isotype control.<br>TIM-3 (RMT3-23) eBioscience (119721): validated by manufacturer: This RMT3-23 antibody has been tested by flow cytometric analysis of mouse splenocytes.<br>MHC Class II (M5/114.15.2) eBioscience (17-5321-82): validated by manufacturer: The M5/114.15.2 antibody has been tested by flow cytometric analysis of mouse splenocytes.<br>GAL-9 (108A.2) Biolegend (137903): raised against Full length recombinant mouse Galectin-9 (M-type), validated by manufacturer by flow cytometric analysis of C57BL/6 thymocytes.<br>CD44 (IM7) Biolegend (103023): raised against Dexamethasone-induced myeloid leukemia M1 cells, validated by manufacturer by flow cytometric analysis of C57BL/6 splenocytes.

CD62L (MEL-14) Biolegend (104426): raised against C3H/eb mouse B lymphoma 38C-13, validated by manufacturer by flow cytometric analysis of C57BL/6 mouse bone marrow cells comparatively stained with an isotype control.
PD-1 (29F.1A12) Biolegend (135219): raised against PD-1 cDNA followed by PD-1-Ig fusion protein, validated by manufacturer by flow cytometric analysis of Con A and IL-2 activated C57BL/6 mouse splenocytes comparatively stained with anti-CD3.
KLRG1 (2F1) Biolegend (138415): raised against IL-2 activated NK cells from C57BL/6 mice, validated by manufacturer by flow cytometric analysis of C57BL/6 mouse splenocytes stained in combination with anti-NK1.1.
DNAM-1 (10E5) Biolegend (128811): raised against Th1 polarized T cell clones, validated by manufacturer by flow cytometric analysis of C57BL/6 mouse splenocytes stained in combination with anti-CD8a and compared to an isotype control.
control IgG (HRPN) Bio-X-Cell (BE0088): The HRPN monoclonal antibody reacts with horseradish peroxidase (HRP). Because HRP is not expressed by mammals this antibody is ideal for use as an isotype-matched control for rat IgG1 antibodies in most in vivo and in vitro applications.
anti-CD4 (GK1.5) Bio-X-Cell (BE0003-1): The GK1.5 monoclonal antibody reacts with the mouse CD4. Validated by manufacturer by western blot against purified mouse CD4.
anti-CD8b (53.5.8) Bio-X-Cell (BE0223): Raised against mouse thymus or spleen. The 53-5.8 antibody has been shown to deplete CD8+ T cells completely but not deplete CD8+ CD11c+ dendritic cells when used in vivo.
anti-PD-1 (RMP1-14) Bio-X-Cell (BE0146): the RMP1-14 antibody has been shown to block the binding of both mouse PD-L1-Ig and mouse PD-L2-Ig to PD-1. Validated by manufacturer by western blotting against purified mouse PD-1.

Eukaryotic cell lines

Policy information about cell lines and Sex and Gender in Research

Cell line source(s)	HEK 293T/17 cells were obtained from ATCC (CRL-11268)
Authentication	HEK 293T/17 cells were validated via STR profiling
Mycoplasma contamination	Cells were tested for mycoplasma contamination prior to expansion and cryopreservation of stock vials which were used for subsequent experiments.
Commonly misidentified lines (See ICLAC register)	n/a

Animals and other research organisms

Policy information about studies involving animals; ARRIVE guidelines recommended for reporting animal research, and Sex and Gender in Research

Laboratory animals	Mus musculus: mice were maintained in a facility with an 8 to 8 light/dark cycle, temperature range of 19-21 degrees Celsius and 55-65% humidity. The Rag2-/-yc-/- strains was maintained on a C57BL/6J background. Donors and recipients were recruited to an experiment between 8-12 weeks of age. Mice were age and sex-matched between groups in all experiments. Npm1c/NrasG12D AML cells were obatined from George Vassiliou via Wallace Langdon. Commercial wild-type C57BL/6J mice were obtained from the Animal Resource Centre (ARC).
Wild animals	This study did not involve wild animals.
Reporting on sex	The majority of experiments used recipient mice of both genders, with inclusion of each gender matched between groups. Information on the gender of animals recruited was recorded but analysis was not performed on a gender-specific basis.
Field-collected samples	This study did not involve field-collected samples.
Ethics oversight	All procedures were performed in accordance with the QIMR Berghofer Animal Ethics Committee (A11605M)

Note that full information on the approval of the study protocol must also be provided in the manuscript.

Flow Cytometry

Plots

Confirm that:

- [x] The axis labels state the marker and fluorochrome used (e.g. CD4-FITC).
- [x] The axis scales are clearly visible. Include numbers along axes only for bottom left plot of group (a 'group' is an analysis of identical markers).
- [x] All plots are contour plots with outliers or pseudocolor plots.
- [x] A numerical value for number of cells or percentage (with statistics) is provided.

Methodology

Sample preparation	Mouse bone marrow was harvested from femurs, tibias and pelvis bones, flushed using a 26 1/2 gauge needle and filtered through a 70 micron filter with PBS, 2% FBS. Mouse spleen and liver cells were isolated from dissected tissues filtered

	through a 70 micron filter using a syringe plunger and PBS, 2% FBS. Peripheral blood was collected from the retro-orbital vein by capillary tube into EDTA-coated vials. All cells were washed in PBS, 2% FBS and red blood cell lysed prior to flow cytometry.
Instrument	BD LSRII Fortessa and BD FACSAriaIII
Software	BD FACS Diva (BD Biosciences) and FlowJo (Tree Star)
Cell population abundance	A minimum of 20, 000 events were collected within post-sort fractions for RNA-Seq samples. Post-sort analysis determined that sample purity was at least over 95%.
Gating strategy	Cells were first gated based on morphology on a FSC-A/SSC-A dot plot to exclude dead cells and debris. FSC threshold was set at 30,000 to avoid collecting these events. GFP positive events in AML-bearing samples were gated based on known GFP-negative samples.

☒ Tick this box to confirm that a figure exemplifying the gating strategy is provided in the Supplementary Information.
