# Supplementary material for: Oncogenic drivers dictate immune control of acute myeloid leukemia
Source: Nat Commun. 2023 Apr 14;14:2155. doi: 10.1038/s41467-023-37592-9 (PMC10104832; doi:10.1038/s41467-023-37592-9)
Supplement: Supplementary file 4 — Description of Additional Supplementary Files [file 41467_2023_37592_MOESM4_ESM.pdf]

Description of Additional Supplementary Files

Supplementary Data 1: Differentially expressed genes and gene set enrichment analysis of NRasG12D and MA9 AML RNA sequencing.

Supplementary Data 2: Gene sets used for analysis in Figure 5D.
